# Practical remediation of the PCB-contaminated soils

**DOI:** 10.1186/s40201-015-0158-2

**Published:** 2015-02-10

**Authors:** Akiko Ido, Miki Niikawa, Shinji Ishihara, Yoshinari Sawama, Tsuyoshi Nakanishi, Yasunari Monguchi, Hironao Sajiki, Hisamitsu Nagase

**Affiliations:** Laboratory of Hygienic Chemistry and Molecular Toxicology, Gifu Pharmaceutical University, 1-25-4 Daigaku-nishi, Gifu, 501-1196 Japan; Laboratory of Organic Chemistry, Gifu Pharmaceutical University, 1-25-4 Daigaku-nishi, Gifu, 501-1196 Japan

**Keywords:** Remediation, PCB-contaminated soil, Soxhlet extraction, Palladium on activated carbon (Pd/C), Hydrodechlorination

## Abstract

A practical method for the elimination of PCBs from PCB-contaminated soil has been developed by the combination of Soxhlet extraction using a newly-developed modified Soxhlet extractor possessing an outlet valve on the extraction chamber with the chemical degradation. Various types of PCBs contaminated in soils could be completely extracted in refluxing hexane, and the subsequent hydrodechlorination could also be completed within 1 h in a hexane–MeOH (1 : 5) solution in the presence of Pd/C and Et_3_N under ordinary hydrogen pressure and temperature without the transfer of the extracted PCBs to other reaction container (a complete one-pot procedure). The present system is quite useful as a simple, safe, mild and reliable remediation method of PCB-contaminated soil.

## Introduction

Polychlorinated biphenyls (PCBs) were commercially produced as mixtures of 209 possible congeners beginning in 1929 through the mid-1970s and are non-polar chlorinated hydrocarbons with a biphenyl nucleus, on which one to ten hydrogen atoms have been replaced with chlorine atoms. PCBs were mainly used as electric insulating oils, lubricants, and coolants in electrical devices, such as transformers and capacitors, all over the world due to their chemical and thermal stabilities. However, their production, import and novel use were banned during the mid-1970s, as such properties led to serious environmental pollution including bioaccumulations and biomagnifications through the food chain [1-4]. While PCBs in general could be decomposed by combustion using high-temperature incinerators (above 1100°C), any drop in temperature below 1100°C creates the possibility to generate more toxic dioxins [5]; hence, it is quite difficult to reach an agreement by local communities for the construction of such incinerators. Therefore, tons of PCBs have been stored by each facility under the strict conditions in accordance with each country’s laws. However, a huge expense and fear of improper disposal and accidental leaks of the PCBs, have increased with the longer periods of storage [6,7].

On the other hand, the fact is that most of the environmental PCB mass has been found in soil including marine, rivered, and lacustrine muck due to their high specific gravity and highly hydrophobic properties [8]. Although many methods for the remediation of PCB-polluted soils have been reported in the literature, such as ultrasonication [9,10], photochemical degradation [11-13], reductive dechlorination using metals [14-16], base-catalyzed decomposition [17-19], hydrogen-transfer hydrodechlorination [20,21], and fungous and bacterial treatments [22-25], most of these remediation methods require severe reaction conditions, such as high heat, high pressure and/or strongly basic conditions, due to the superior chemical stability of PCBs and the difficulty of complete extraction from the contaminated soil due to the strong affinity between the PCBs and soil based on a hydrophobic interaction. While direct methods for the PCB degradation without the PCB-extraction process from soil have been reported [13,18,19,22-25], a PCB extraction process was involved in most remediation parts for the reliable and reproducible PCB clean up using chemical degradation methods [9-11,14-16]. PCBs are basically extracted from their polluted soils by the separatory funnel method using dichloromethane [16] or hexane–acetone [15], the ultrasonic extraction method in a hexane–acetone mixed solvent [14], and the Soxhlet extraction method [11,26-28]. The United States Environmental Protection Agency (U.S. EPA) recommends Soxhlet extraction using hexane and acetone (1 : 1, v/v) as a standard method to isolate PCBs from soil samples [26,27]. The Soxhlet extraction using toluene as a solvent was adopted as an official method to survey the dioxin concentration including co-planar PCBs in soil samples by the Ministry of the Environment of the Japan government [28].

We recently established an efficient method for the palladium on carbon (Pd/C)-catalyzed hydrodechlorination of aromatic chlorides using triethylamine (Et_3_N) as a single electron donor at ambient temperatures and pressures [29,30], which has been successfully used for the degradation of dichlorodiphenyltrichloroethanes (DDTs) [31] and PCBs [32,33] based on the removal of the chlorine atoms from the aromatic nuclei, and a pilot study using a 50 L vessel for the practical PCB degradation was achieved [34]. Furthermore, magnesium metal was also used as a single electron donor instead of Et_3_N for the Pd/C-catalyzed PCB degradation methods [35,36].

In this paper, we describe an easy and reliable remediation method of PCB-polluted soils by PCB extraction and continuous Pd/C–Et_3_N-mediated complete hydrodechlorination of the extracted PCBs.

## Methods

### Materials

Quartz sand, diatomaceous earth, and bentonite were purchased from Nacalai Tesque, Inc. (Kyoto, Japan), and the silica sand (30–50 mesh) was purchased from Koso Chemical Co., Ltd. (Tokyo, Japan). The soil samples, Akadama-tsuchi and Kanuma-tsuchi, are clean, nonpollutant, mildly acidic, and produced in Tochigi prefecture, Japan. They were purchased as 2 L packs from Tachikawa Heiwa Nouen Co., Ltd. (Tochigi, Japan). The soil was ground by a mortar and pestle before use. Also no contamination by PCBs of all purchased soil samples (quartz sand, diatomaceous earth, bentonite, silica sand, Akadama-tsuchi and Kanuma-tsuchi) was confirmed by gas chromatography/mass spectrometry (GC/MS) analysis after Soxhlet extraction using hexane as an extraction solvent.

Aroclor 1242 was previously manufactured by the Monsanto Chemical Co. (St. Louis, MO). 10% Pd/C (K-type, wet), which contained approximately 50% H_2_O, and Et_3_N were obtained from N.E. Chemcat Co., Ltd. (Tokyo, Japan) and Wako Pure Chemical Industries, Ltd. (Osaka, Japan), respectively. Both hexane and MeOH were purchased from Nakalai Tesque, Inc. [Product number: 04338–93 (hexane) and 04338–41 (MeOH), Kyoto, Japan]. All other reagents were purchased from commercial sources and used without further purification.

**Figure 1:**
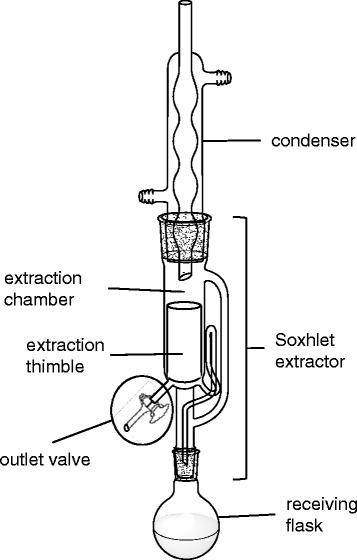
**Modified Soxhlet extractor for the extraction of PCBs in soil.**

A Soxhlet extractor, which has an outlet valve on the extraction chamber, was designed for the easy removal of the collected (distilled) solvents without taking apart the Soxhlet extraction setup (Figure 1).

### Preparation of PCB-contaminated soils

A diethyl ether solution of Aroclor 1242 (1 mg/mL) was spread evenly to the purchased typical soil (quartz sand, silica sand, diatomaceous earth, bentonite, Akadama-tsuchi or Kanuma-tsuchi) to prepare the PCB-contaminated soils (250 μg PCBs/g). Each PCB-contaminated soil was stored at 35°C (oil bath) for 0.5–1 h to allow evaporation of the diethyl ether.

### PCB extraction from PCB-contaminated soils using Soxhlet extractor

**Figure 2:**
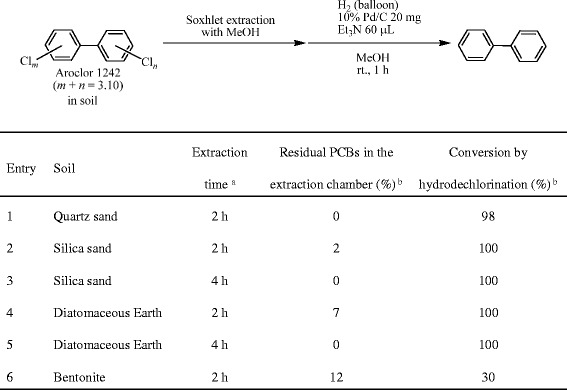
**PCB extraction from PCB-contaminated soils with MeOH and hydrodechlorination under 10% Pd/C–Et**_**3**_ **N–H**_**2**_ **conditions. a** Using a modified Soxhlet extractor (Figure 1) **b** Determined by GC/MS.

Figure 2, Entry 1: The Soxhlet extraction of PCBs from the PCB-contaminated quartz sand (30 g), which was placed in the extraction thimble, was conducted using MeOH (150 mL) as an extraction solvent for 2 h using a modified Soxhlet extractor that has an outlet valve connected on the extraction chamber for the easy removal of the collected (distilled) solvents after completion of the extraction (Figure 1). The PCB extract in the receiving flask was then heated at 90°C, and the distilled MeOH (ca. 35 mL) was collected in the extraction chamber and removed through the outlet valve. The removed MeOH solution (1.5 mL) was concentrated to dryness, and the residue was dissolved in hexane (1.5 mL) and analyzed by GC/MS. The residual PCBs in the extraction chamber were estimated based on the comparison of the total peak area of the PCBs between the PCB contents in the original PCB-contaminated soils and the PCB contents in the recovered soils after extraction. A part (1.5 mL) of the Soxhlet extract in the receiving flask [MeOH solution (100 mL)] was also concentrated to dryness and dissolved in hexane (1.5 mL). The hexane solution (800 μL) was diluted with additional hexane (400 μL), and the GC/MS analysis was conducted to confirm the recovery of the Aroclor 1242 in the receiving flask. The recovery ratio of Aroclor 1242 was estimated to be 93% by comparison of the peak area of the prepared hexane sample using the recovered PCBs in MeOH in the receiving flask with the PCBs in the original PCB-contaminated quartz sand (7.5 mg).

Figure 2, Entry 2: The same procedure used in Entry 1 was followed except for using silica sand (30 g) in place of the quartz sand (30 g).

Figure 2, Entry 3: The same procedure used in Entry 2 was followed, and the extraction time was extended to 4 h. The silica sand in the extraction thimble was recovered and extracted again with fresh hexane (150 mL) using the Soxhlet extractor for 2 h. No residual PCBs in the extraction chamber and receiving flask were detected by GC/MS.

Figure 2, Entry 4: The procedure was the same as the method in Entry 1 except for using diatomaceous earth (10 g) in place of the quartz sand (30 g). The amount of soil was determined according to its bulkiness due to the limited volume of the extraction thimble.

Figure 2, Entry 5: The same procedure used in Entry 4 was followed, and the extraction time was extended to 4 h. The diatomaceous earth in the extraction thimble was recovered and extracted again with fresh hexane (150 mL) using the Soxhlet extractor for 2 h. No residual PCBs in the extraction chamber and receiving flask were detected by GC/MS.

Figure 2, Entry 6: The procedure was the same as the method in Entry 1 except for using bentonite (20 g) in place of the quartz sand (30 g).

**Figure 3:**
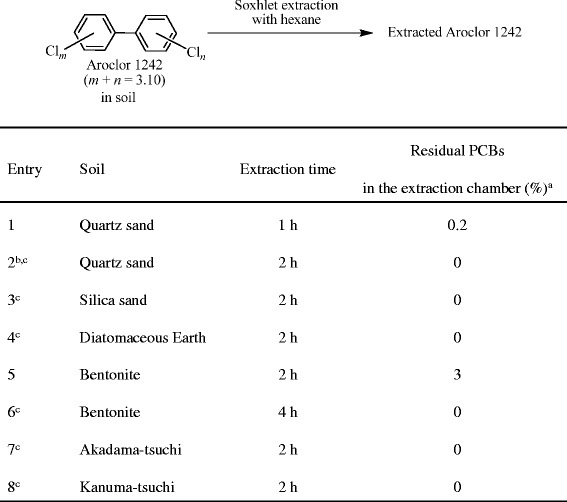
**PCB extraction from PCB-contaminated soils with hexane using a modified Soxhlet extractor. a** Determined by GC/MS. **b** The extraction and hydrodechlorination of PCBs were investigated three times. **c** The extracted PCBs in the receiving flask were used for the hydrodechlorination, see Figure 7.

Figure 3, Entry 1: The extraction procedure was the same as the method in Figure 2, Entry 1 except for the use of hexane (150 mL) as an extraction solvent and a 1-h extraction time. The PCB extract in the receiving flask was heated at 90°C, and the distilled hexane (ca. 35 mL) was collected in the extraction chamber and removed through the outlet valve. The removed hexane solution (1 μL) from the extraction chamber was analyzed by GC/MS without dilution.

Figure 3, Entry 2: The extraction procedure was the same as the method for Figure 3, Entry 1 except for the extraction time (2 h).

Figure 3, Entry 3: The extraction procedure was the same as the method for Figure 3, Entry 2 except for the use of silica sand (30 g) in place of the quartz sand (30 g).

Figure 3, Entry 4: The extraction procedure was the same as the method for Figure 3, Entry 2 except for the use of diatomaceous earth (10 g) in place of the quartz sand (30 g).

Figure 3, Entry 5: The extraction procedure was the same as the method for Figure 3, Entry 2 except for the use of bentonite (20 g) in place of the quartz sand (30 g).

Figure 3, Entry 6: The extraction procedure was the same as the method for Figure 3, Entry 1 except for the extraction time (4 h) and use of bentonite (20 g) in place of the quartz sand (30 g).

Figure 3, Entries 7 and 8: The extraction procedure was the same as the method for Figure 3, Entry 2 except for the use of Akadama-tsuchi (20 g) (Entry 7) or Kanuma-tsuchi (20 g) (Entry 8) in place of the quartz sand (30 g).

In Figure 3, Entries 2–4 and 6–8, the Soxhlet extracts in the receiving flask was used for the PCB degradation under the 10% Pd/C–Et_3_N–H_2_ conditions using the procedure described in the method section “Hydrodechlorination of PCB extracts from PCB-polluted soils under 10% Pd/C–Et_3_N–H_2_ conditions”.

### The solvent effect on the hydrodechlorination of the PCBs under 10% Pd/C–Et_3_N–H_2_ conditions

**Figure 4:**
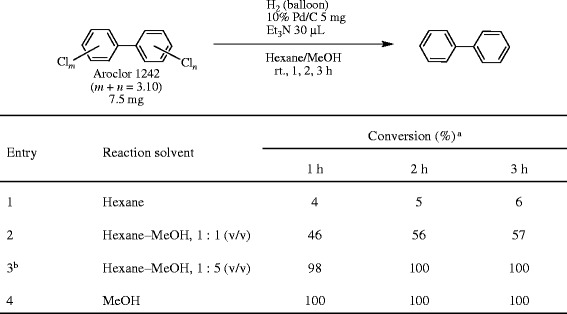
**Solvent effect on the hydrodechlorination of PCBs under 10% Pd/C–Et**_**3**_ **N–H**_**2**_ **conditions. a** Determined by GC/MS. **b** The GC charts after 1, 2, and 3-h reactions are shown in Figure 5.

Figure 4, Entry 1: A mixture of Aroclor 1242 (7.5 mg), 10% Pd/C (5 mg) and Et_3_N (30 μL) in hexane (60 mL) in a round-bottom flask was vigorously stirred using a stirring bar under a hydrogen atmosphere (balloon) at ambient temperature (ca. 25°C). After 1, 2, and 3 h, the reaction mixture (1 mL) was sampled, filtered through a 0.45 μm membrane filter (Millipore, USA), and washed with H_2_O (1 mL). The conversion yield (%) was determined by GC/MS analysis of the resulting hexane solution.

Figure 4, Entry 2: The procedure was the same as the method for Figure 4, Entry 1 except for the solvent [hexane–MeOH (1 : 1, 60 mL)] and the amount of the sampled reaction mixture (1.5 mL), which was successively washed with H_2_O (1.5 mL). The conversion yield (%) was determined by GC/MS analysis using the doubly diluted hexane layer.

**Figure 5:**
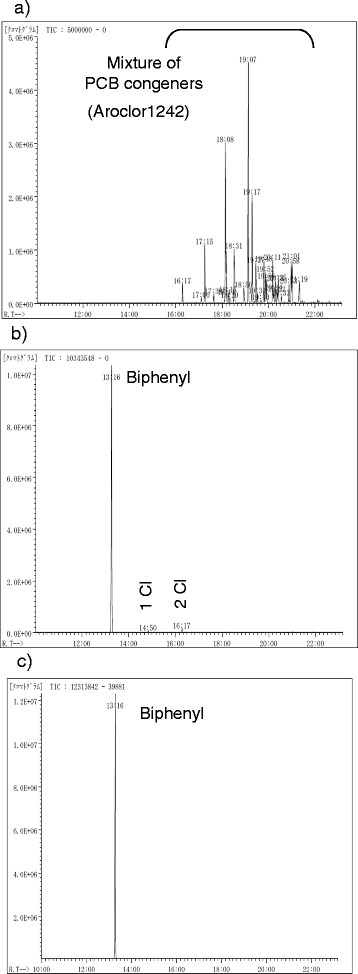
**GC/MS charts for the time-course of the dechlorination of Aroclor 1242.** The dechlorination of Aroclor 1242 was carried out in hexane–MeOH (1 : 5) for **a)** 0 h, **b)** 1 h and **c)** 2 h under the Pd/C-Et_3_N-H_2_ combination (Figure 4, Entry 3).

Figure 4, Entry 3: The procedure was the same as the method for Figure 4, Entry 1 except for the solvent [hexane–MeOH (1 : 5, 60 mL)] and the amount of the sampled reaction mixture (1.5 mL). Hexane (1.5 mL) and H_2_O (1.5 mL) were added to the sampled reaction mixture, the mixture was shaken, then the hexane layer was used for the GC/MS analysis. The GC charts after 0, 1, and 2 h are shown in Figure 5.

Figure 4, Entry 4: The procedure was the same as the method for Figure 4, Entry 1 except for the solvent [MeOH (60 mL)]. The reaction mixture (1.5 mL) was sampled, filtered, and concentrated to dryness, then the residue was dissolved in hexane (1 mL). The solution was washed with H_2_O (1 mL) and the hexane layer was used for the GC/MS analysis after a double dilution.

### Evaluation of residual PCBs in distilled hexane in the extraction chamber after the Soxhlet extraction

**Figure 6:**
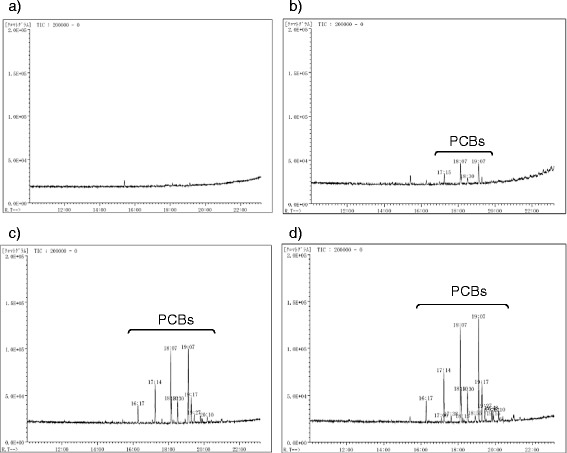
**GC spectra of the collected hexane in the extraction chamber after the 2 h-Soxhlet extraction. a)** first fraction: distilled hexane collected in the extraction chamber; **b)** second fraction: distilled hexane collected in the extraction chamber from the remaining hexane solution in the receiving flask after the removal of the first fraction; **c)** third fraction: distilled hexane from the remaining hexane solution in the receiving flask after the removal of the second fraction; **d)** fourth fraction: distilled hexane from the remaining hexane solution in the receiving flask after the removal of the third fraction.

The Soxhlet extraction of PCBs from the PCB-contaminated quartz sand (30 g) using hexane (150 mL) was carried out for 2 h. The extract in the receiving flask was concentrated at 90°C. The distilled and collected hexane solution (ca. 30 mL, first fraction) was removed from the extraction chamber through the outlet valve (Figure 1), and the first fraction (1 μL) was analyzed by GC/MS (Figure 6a). The remaining extract in the receiving flask was gently and further concentrated at 90°C, the distilled and collected hexane in the extraction chamber (ca. 30 mL) was removed through the outlet valve as the second fraction, and its GC/MS analysis (1 μL) was also performed (Figure 6b). Two more hexane fractions (third and fourth fractions, each ca. 30 mL) were collected in the extraction chamber from the extracts in the receiving flask by heating at 90°C, removed through the outlet valve, and analyzed using GC/MS in the same manner as described for the first and second fractions (Figure 6c and d).

### Hydrodechlorination of PCB extracts from PCB-polluted soils under 10% Pd/C–Et_3_N–H_2_ conditions

Figure 2: 10% Pd/C (20 mg) and Et_3_N (60 μL) were added to the PCB extract in MeOH (ca. 100 mL) in the receiving flask (see the method section for Figure 2, “PCB extraction from PCB-contaminated soils using Soxhlet extractor”). The mixture was vigorously stirred using a stirring bar under a hydrogen atmosphere (balloon) at ambient temperature (ca. 25°C) for 1 h. The reaction mixture (ca. 5 mL) was filtered through cotton, and the filtrate (3 mL) was concentrated to dryness. The residue was diluted with hexane (3 mL) and washed with H_2_O (3 mL). The hexane layer (800 μL) was diluted with additional hexane (400 μL), and the resulting hexane solution was analyzed by GC/MS to analyze the degradation.

**Figure 7:**
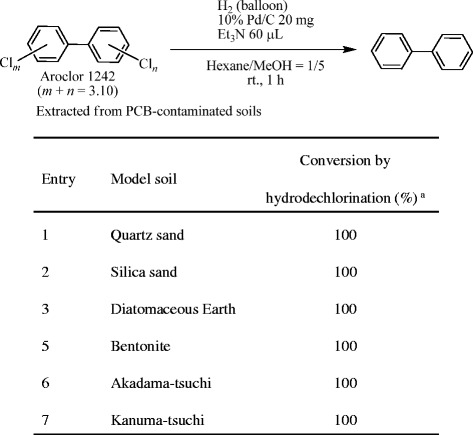
**Hydrodechlorination of the extracted PCBs under 10% Pd/C–Et**_**3**_ **N–H**_**2**_ **conditions. a** Determined by GC/MS.

**Figure 8:**
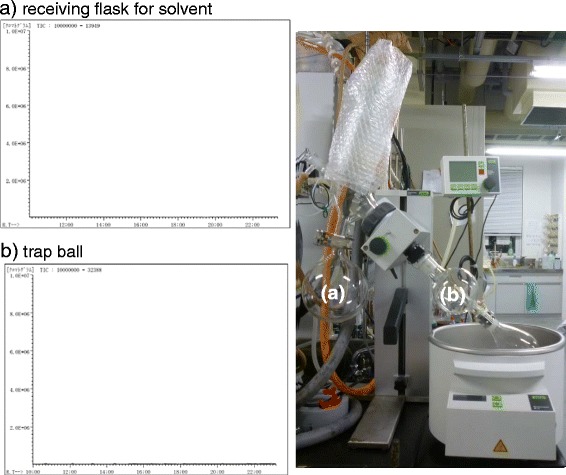
**The GC/MS charts of the recovered hexane in the evaporator after the concentration.** The GC/MS charts of the recovered hexane **a)** in the receiving flask (shown by (a) in the panel) and **b)** in the trap ball (shown by (b) in the panel) of the rotary evaporator. The panel shows rotary evaporator used in this study.

Figure 7: Each extracted PCB in hexane in the receiving flask (ca. 100 mL, see the method section for Figure 3, Entries 2–4 and 6–8, “PCB extraction from PCB-contaminated soils using Soxhlet extractor”) was very gently concentrated to ca. 10 mL using a rotary evaporator (40°C, ca. 20 mmHg), and no PCBs were detected in the hexane collected in the rotary evaporator trap [b) in Figure 8] and receiving flask [a) in Figure 8] by GC/MS. MeOH (50 mL), 10% Pd/C (20 mg), and Et_3_N (60 μL) were added to the receiving flask. The mixture was vigorously stirred using a stirring bar under a hydrogen atmosphere (balloon) at ambient temperature (ca. 25°C) for 1 h. The reaction mixture (ca. 6 mL) was filtered through a 0.45 μm membrane filter (Millipore, USA) and concentrated to dryness. The residue was diluted with hexane (5 mL) and washed with H_2_O (5 mL). The hexane layer (400 μL) was diluted with additional hexane (600 μL), and the resulting hexane solution was analyzed by GC/MS to analyze the degradation.

### Instrumental analysis

A JMS Q1000 GC [7890A gas chromatography (Agilent Technologies, USA) equipped with a JEOL MK II mass spectrometer (JEOL Co., Ltd., Japan)] and an Inert Cap 5MS/sil + GD capillary column (30 m × 0.25 mm i.d., 0.25 μm film thickness: GL Science, Japan) were used for the PCB analysis. Helium was employed as a carrier gas at the flow rate of 1.3 mL/min. The injector and detector temperatures were 280°C. The column temperature was programmed to ramp from 70°C (5 min hold) to 280°C (4 min hold) to the rate of 10°C/min. One μL of the sample solution was injected. The retention time of biphenyl was 13.01 min and those of the PCBs (Aroclor 1242) were 16.26–21.29 min. The products were identified by their GC/MS retention times in comparison to those of authentic commercial samples.

## Results and discussion

### Soxhlet extraction of PCBs from PCB-contaminated soil

PCBs were extracted from a variety of artificially PCB-contaminated soils (quartz sand, silica sand, diatomaceous earth and bentonite) for 2 h using a modified Soxhlet extractor that has an outlet valve attached to the extraction chamber for the easy removal of the extraction solvent (Figure 1). MeOH was first examined as an extraction solvent for the present Soxhlet extraction (Figure 2), since MeOH was found to be an effective and preferable solvent for the Pd/C-catalyzed PCB degradation based on the dechlorination reaction in the presence of Et_3_N under an H_2_ atomosphere [29,30,32,33]. Thus, the use of MeOH for the extraction could avoid the solvent exchange process for the PCB degradation after the extraction. When the 2-h Soxhlet extraction of the PCBs from the PCB-contaminated quartz sand was carried out, no PCBs were detected from the MeOH collected in the extraction chamber (Entry 1), and the nearly quantitative extraction of the PCBs was confirmed by the GC/MS analysis of the PCBs in the receiving flask (93%). We determined that the PCBs were nearly quantitatively extracted in the receiving flask when no PCBs were detected in the solvents obtained from the extraction chamber after the Soxhlet extraction. Although the MeOH extract in the receiving flask was vigorously stirred with 10% Pd/C and Et_3_N under an H_2_ atmosphere (balloon) at room temperature (ca. 25°C) for 1 h, the hydrodechlorination of the PCBs in the extract was not completed (98% conversion) contrary to our expectation. The incomplete reaction would be attributed to the sulfur contaminants in the original quartz sand, since significant amounts of sulfur contaminants [1800 μg/g by the oxygen bomb combustion ion chromatography (Sumika Chemical Analytical Service, Ltd., Japan)] were extracted by MeOH from the quartz sand. Such sulfur components could function as catalyst poisons toward the Pd/C-catalyzed hydrodechlorination based on the significant coordination activity of sulfur atoms to palladium atoms. Therefore, MeOH was not an appropriate solvent for the Soxhlet extraction from the PCB-polluted quartz sand.

Although a small amount of PCBs was still extracted in MeOH in the extraction chamber after the 2-h extraction of the PCB-contaminated silica sand (2%, Figure 2, Entry 2) and diatomaceous earth (7%, Entry 4), the lengthening of the extraction time to 4 h certainly enhanced the extraction efficiency, and no PCBs were detected in MeOH in the extraction chamber (Entries 3 and 5). The soils in the extraction thimble after 4-h extraction (Entries 3 and 5) were recovered, and further extraction was conducted with fresh hexane using the Soxhlet extractor for 2 h, since hexane was generally employed as an effective solvent for the PCB extraction [11,14]. Consequently, no PCBs were detected from both the extraction chamber and the receiving flask, indicating that the PCBs were completely extracted from soils by the first 4-h Soxhlet extraction. Therefore, we estimated that the complete PCB extraction was achieved when no PCBs were detected in the solvent in the extraction chamber. Furthermore, PCBs in the MeOH extracts of the PCB-contaminated silica sand and diatomaceous earth underwent a complete degradation under Pd/C–Et_3_N-mediated hydrodechlorination conditions (Entries 3 and 5). On the other hand, the Soxhlet extraction of the PCB-contaminated bentonite using MeOH was unsuccessful probably due to the strong adsorptive property, affinity for MeOH and/or viscosity of the bentonite, and a significant amount of PCBs (12%) still remained in MeOH in the extraction chamber after 2-h extraction. Furthermore, the subsequent 10% Pd-catalyzed hydrodechlorination proceeded in only 30% (Entry 6). Bentonite is originally derived from volcanic ash and contains a substantial amount of sulfur concomitants, such as sulfates and sulfides. The sulfur materials would be extracted from the bentonite together with PCBs by the Soxhlet extraction with MeOH and strongly suppress the PCB degradation as catalyst poisons in analogy (more significantly) with the hydrodechlorination of the MeOH extracts of quartz sand (Entry 1). Therefore, we concluded that MeOH was not effective as a solvent for the Soxhlet extraction of PCBs.

Hexane has been mainly used as an efficient solvent for the extraction of PCBs from the PCB-contaminated solid materials and water in the literature [11,14,37,38]. Furthermore, the low solubility of sulfur contaminants in hexane is also easily predicted compared to MeOH. Therefore, the Soxhlet extraction of PCBs with hexane from the PCB-contaminated quartz sand was carried out to establish the effective PCB extraction method (Figure 3, Entries 1 and 2). Although the 1-h Soxhlet extraction was incomplete for the perfect removal of PCBs from the PCB-contaminated quartz sand and 0.2% of the PCBs still remained in hexane in the extraction chamber (Entry 1), the PCB-residue completely disappeared from the extraction chamber by extending the extraction time to 2 h (Entry 2). The Soxhlet extraction procedure for PCBs with hexane was reliable since it could be reproduced three times (Entry 2), and the amounts of the extracted sulfur contaminants from the PCB-contaminated quartz sand were notably decreased compared to the extraction with MeOH [880 μg/g by oxygen bomb combustion ion chromatography (Sumika Chemical Analytical Service, Ltd., Japan)]. These results indicated that the PCBs were completely and selectively extracted from the polluted quartz sand by hexane. The Soxhlet extraction method using hexane could also be applied to extraction from the PCB-contaminated silica sand and diatomaceous earth (Entries 3 and 4), although the extraction of PCB-contaminated bentonite was incomplete within 2 h (Entry 5) probably because bentonite could strongly adsorb PCBs due to its strong viscosity property. A quantitative extraction of PCBs from PCB-contaminated bentonite was successfully achieved by extending the extraction time to 4 h (Entry 6).

The method for the remediation of PCB-polluted soils was also used for the cleanup of horticultural soils, Akadama-tsuchi and Kanuma-tsuchi (Entries 7 and 8). The PCBs in both the PCB-contaminated Akadama-tsuchi (Entry 7) and Kanuma-tsuchi (Entry 8) were completely extracted with hexane within 2 h using the modified Soxhlet extractor.

The extracted Aroclor 1242 in the receiving flask from polluted-quartz sand (Entry 2), −silica sand (Entry 3), −diatomaceous earth (Entry 4), −bentonite (Entry 6), −Akadama-tsuchi (Entry 7), and -Kanuma-tsuchi (Entry 8) were successively employed for the Pd/C-catalyzed hydrodechlorination, which will be discussed later (Figure 7).

### Solvent effect on the PCB dechlorination

Since hexane was found to be appropriate as a solvent for the Soxhlet extraction of PCBs, the use of hexane as a solvent for the degradation of PCBs based on the hydrodechlorination of aromatic chloride under Pd/C–Et_3_N–H_2_ conditions would be desired due to the safe and easy handling on the basis of no need to exchange solvents after the extraction. However, the 10% Pd/C-catalyzed hydrodechlorination of Aroclor 1242 in the presence of Et_3_N hardly proceeded in hexane (Figure 4 Entry 1). The detailed study of the Pd/C-catalyzed hydrodechlorination in the presence of Et_3_N under an H_2_ atmosphere has already revealed that MeOH was an effective and preferable solvent [29,30,32,33]. Aroclor 1242 was completely hydrodechlorinated at room temperature within 1 h in MeOH instead of hexane (Entry 4). The mixed solvent of hexane and MeOH was then investigated, since the complete removal of hexane from the receiving flask for the exchange of solvents after the Soxhlet extraction includes the risk of the possible evaporation or leak of PCBs together with hexane (see the discussion in the section “Procedure for the concentration of the PCB extract in the receiving flask”). The higher MeOH ratio of the mixed solvents more effectively promoted the hydrodechlorination (Figure 4 Entries 2 and 3), and it could be completed in hexane–MeOH (1 : 5) within 2 h under ambient pressures and temperatures (Entry 3) as shown in Figure 5.

### Concentration of the PCB extract in the receiving flask

We next investigated the establishment of a procedure for the removal of hexane from the Soxhlet extract in the receiving flask for the subsequent Pd/C-catalyzed hydrodechlorination of PCBs in hexane–MeOH (1 : 5). After the 2-h Soxhlet extraction of PCBs from the PCB-contaminated quartz sand using hexane, the hexane was transferred to the extraction chamber from the receiving flask by the four-step distillation, and collected as different fractions (Figure 6). Although no PCBs were detected in the first fraction (Figure 6a), a small quantity of PCBs was found in the 2nd fraction (Figure 6b), and the detected PCB quantity increased in a fraction-number-dependent manner (Figure 6b–d). These results suggest that a small amount of PCBs could be vaporized together with the distilled hexane with decreasing amount of hexane in the receiving flask even under gentle refluxing conditions. On the other hand, when the further gentle concentration of the hexane extract in the receiving flask using a rotary evaporator under reduced pressure (ca. 20 mmHg) at 40°C was done, no PCBs were detected in the collected hexane in both the rotary evaporator trap [b) in Figure 8] and the solvent receiving flask [a) in Figure 8] by GC/MS, indicating that no PCBs had been vaporized during the gentle evaporation under a sufficiently reduced pressure.

### Continuous operation of the PCB extraction using the Soxhlet extractor and dechlorination using the 10% Pd/C–Et_3_N–H_2_ conditions

The PCB extraction from a variety of PCB-contaminated soils (quartz sand, silica sand, diatomaceous earth, bentonite, Akadama-tsuchi and Kanuma-tsuchi) with hexane (Figure 3) and the subsequent dechlorination using Pd/C–Et_3_N–H_2_ conditions in hexane–MeOH were carried out under ambient H_2_ pressures (balloon) and temperatures (Figure 7). While the PCBs in the MeOH extract from the PCB-contaminated quartz sand were not completely hydrodechlorinated under 10% Pd/C–H_2_–Et_3_N conditions (Figure 2, Entry 1), the degradation of the hexane extract (Figure 3, Entry 2) was completed (Figure 7, Entry 1) because the amount of sulfur-contaminants in the hexane extract was sufficiently low (880 μg/g) compared to that of MeOH (1800 μg/g). Furthermore, all other PCB extracts derived from the silica sand, diatomaceous earth, bentonite, Akadama-tsuchi and Kanuma-tsuchi were also smoothly and completely degraded within 1 h (Figure 7, Entries 2–7).

The present soil remediation method using a modified (newly-developed) Soxhlet extractor that bears an outlet valve on the extraction chamber is able to extract PCBs from PCB-contaminated soils completely and the subsequent chemical degradation is successfully accomplished by a one-pot procedure. In reported remediation methods in the literature involved extraction process, the PCB extraction from soil and its chemical degradation could be achieved totally independent manner with the exchange of the reaction containers [9-11,14-16], to our knowledge. The present method is quite safe by reason that the operators could prevent the extracted PCBs from the direct contacting. Furthermore, PCBs extracted from PCB-contaminated soils quantitatively degrade based on a Pd/C-catalyzed dechlorination in the presence of Et_3_N under ambient hydrogen pressure and temperature in a short time. Moreover, the Pd/C might be recovered and reused by the simple wash in water and hexane sequentially [32]. The present remediation method achieved extensive improvement in simplicity, safety and time-efficiencies over the previously reported remediation processes.

## Conclusions

We developed a practical method for the remediation of PCB-contaminated soils. One of the notable features of the PCB remediation of PCB-contaminated soils is the use of a receiving flask for the Soxhlet extractor as a reaction flask for the hydrodechlorination of PCBs. Therefore, the present remediation method never requires the transfer of the extracted PCBs to another reaction container and the degradation is successfully accomplished by a one-pot procedure. Thus, the possible secondary pollution by the extracted (concentrated) PCBs does not occur. PCBs are efficiently extracted with hexane from various types of polluted soils, and their dechlorination could be easily achieved in a hexane–MeOH (1 : 5) solution using the 10% Pd/C–Et_3_N–H_2_ conditions under ambient pressures (balloon) and temperatures (ca. 25°C). The present method is quite simple and would be useful for the mild and practical remediation of PCB-polluted soils.

## Abbreviations

PCBs: Polychlorinated biphenyls
Pd/C: Palladium on activated carbon
Et_3_N: Trimethylamine
U.S.EPA: The United States environmental protection agency
DDTs: Dichlorodiphenyltrichloroethanes
GC/MS: Gas chromatography/mass spectrometry
